# Green *Coffea arabica* Extract Ameliorates Testicular Injury in High-Fat Diet/Streptozotocin-Induced Diabetes in Rats

**DOI:** 10.1155/2020/6762709

**Published:** 2020-06-12

**Authors:** Wafa A. AL-Megrin, Manal F. El-Khadragy, Manal H. Hussein, Shahenda Mahgoub, Doaa M. Abdel-Mohsen, Heba Taha, Ashraf A. A. Bakkar, Ahmed E. Abdel Moneim, Hatem K. Amin

**Affiliations:** ^1^Biology Department, Faculty of Science, Princess Nourah Bint Abdulrahman University, Riyadh 11671, Saudi Arabia; ^2^Zoology and Entomology Department, Faculty of Science, Helwan University, Cairo, Egypt; ^3^Biochemistry and Molecular Biology Department, Faculty of Pharmacy, Helwan University, Cairo, Egypt; ^4^Faculty of Biotechnology, Modern Sciences and Arts University (MSA), Giza, Egypt

## Abstract

Diabetes mellitus (DM) is a chronic endocrine disease characterized by persistent hyperglycemia. Oxidative damage, inflammatory cytokines, and apoptotic cell death play a major role in the induction and progression of male testicular damage. Plant-derived phytochemicals such as green coffee (*Coffea arabica*) can possess antidiabetic effects with little toxicity. The current study is aimed at investigating the therapeutic roles of green coffee in diabetic testicular injury stimulated by high-fat diet/streptozotocin administration. Diabetes mellitus was induced by a high-fat diet and a single dose of streptozotocin (STZ) (35 mg kg^−1^) in male albino rats. Diabetic animals were orally given two different concentrations of green coffee (50 mg kg^−1^ and 100 mg kg^−1^) for 28 days. The levels of testosterone, luteinizing hormone, and follicle-stimulating hormone and parameters of oxidative stress, inflammation, and apoptosis were measured. mRNAs and protein levels were detected quantitatively by real-time PCR and ELISA, respectively. In the diabetic group, the levels of testosterone, luteinizing hormone, and follicle-stimulating hormone showed a significant reduction while they increased significantly after green coffee treatment. A significant increase of antioxidant markers glutathione, superoxide dismutase, catalase, glutathione peroxidase, and glutathione reductase along with decreased levels of lipid peroxides and nitric oxide was observed after green coffee treatment in the diabetic group. Finally, the levels of IL-1*β*, TNF-*α*, Bax, and caspase-3 were also decreased in both treated groups (metformin and green coffee) when compared to the diabetic group. We conclude that testicular oxidative impairment induced by a high-fat diet (HFD) and STZ can be reversed by green coffee. Administration of green coffee could represent a promising therapeutic agent which can help the treatment of type 2 DM-induced testicular dysfunction.

## 1. Introduction

Diabetes is a long-lasting endocrine disorder described as persistent hyperglycemia, which is often triggered by the entire or relative shortage of insulin production or insulin resistance [1]. A wealth of reports indicated that oxidative stress [2], inflammation [3], and apoptosis [4] are responsible for the generation of testicular injury in males. Such complications include testicular weight loss, defective spermatogenesis, and germ cell death along with altered levels of reproductive hormones and accumulation of fat droplets [3].

At present, available diabetic therapy achieved some success which includes insulin and multiple oral hypoglycemic medicines. However, many of these drugs have some side effects [5]. Antihyperglycemic drugs such as metformin can regulate the glycemic mechanism to some extent, but these drugs do not improve the *β*-cell dysfunction in diabetic patients [6]. Therefore, it is necessary to find a natural-based therapy which can help reduce testicular injury in diabetic patients. Plant-derived phytochemicals can possess antidiabetic effects by interfering with glucose metabolism with little toxicity or minimal side effects [7].

Chlorogenic acid (CGA) is the most important bioactive component in green coffee (*Coffea arabica*) [8]. Some previous studies on experimental animals and humans suggested that the therapeutic properties of green coffee are linked to the antioxidant and anti-inflammatory capacities of bioactive CGA [9, 10]. Interestingly, CGA can reduce body fat mass and plasma lipid profile in high-fat diet-induced obese mice [11]. Moreover, CGA reduces insulin resistance and diminishes the hyperglycemic levels targeting hepatic glucose metabolism [12]. Additionally, the previous *in vitro* studies demonstrated the protective effects of *Coffea arabica* extract against oxidation and DNA injury in the human and animal cell models with no cytotoxicity. In this regard, Affonso et al. [13] found that the LD_50_ value of roasted coffee beans using mouse L929 fibroblasts was 2482.00 mg ml^−1^. Jung et al. [14] observed that coffee extract has no toxicity on the viability of AML-12 and RAW 264.7cells until 2.0 mg ml^−1^.

Streptozotocin (STZ) causes apoptosis of *β*-cells by DNA alkylation and production of oxidative markers resulting in impaired insulin-glucose homeostasis and ultimately diabetes [4]. A high-fat diet and low-dose single injection of STZ were adopted to induce T2DM in rats according to previous studies [15].

Previously, Moustafa [16] studied the effect of green coffee on the histological and ultrastructural alterations in testes of obese rats. However, no study investigated the effect of green coffee on oxidative stress, inflammation, and apoptosis markers in testes of rats that suffered from T2DM. Hence, the current study was designed to evaluate the therapeutic effects of green coffee in diabetes mellitus induced by high-fat diet/streptozotocin. We evaluated its role in the modulation of oxidative stress, in the inflammatory immune response, and as an antiapoptotic agent to reduce the induced testicular damage.

## 2. Material and Methods

### 2.1. Plant Material

Green *Coffea arabica* beans were obtained from a local seller in East Cairo, Egypt, in October 2018. The beans were identified and authenticated by a specialist (Botany Department, Faculty of Science, Helwan University, Egypt). The green coffee beans were ground into fine powder using an electrical blender. The obtained powder was soaked in water (70°C) for 20 min similar to the traditional way of preparing a coffee drink by Arabians. After that, the extract was filtered and lyophilized to remove water. The final physical appearance of the extract was powder. The extract was defined as a GCWE (green coffee water extract), dissolved in water to be 100 mg/ml, and stored at −20°C until use.

### 2.2. Determination of Total Phenolics and Flavonoids

The total phenolic (TP) and flavonoid (TF) levels were estimated by the Folin–Ciocalteu and AlCl_3_ methods, respectively, as previously described by Abdel Moneim [17]. The TP and TF levels were expressed as milligram of gallic acid equivalents per gram of GCWE dried weight (mg GAE/g GCWE DW) and milligram of quercetin equivalents per gram of GCWE dried weight (mg QE/g GCWE DW) using the calibration curves of gallic acid and quercetin, respectively.

### 2.3. Animals

A total of forty-two male rats weighing about 200-220 g were purchased from VACSERA (Cairo, Egypt). All rats were maintained in a chamber under a suitable environment of temperature 25 ± 5°C and humidity 50 ± 10% with alternating light and dark cycles of 12 h and acclimated for 1 week with unrestricted water and food prior to the inception of experiments. All procedures of the experimental model were in agreement with the guiding principles of the Institutional Animal Care and Use Committee (IACUC) of Helwan University (approval no. HU2018/Z/AE0518-010).

### 2.4. Induction of Type 2 Diabetes Mellitus in Experimental Animals

Diabetes mellitus (DM) was induced by feeding the rats a high-fat diet (HFD) (total energy 25.07 kJ/g including fat 60%, protein 20%, and carbohydrate 20%) for 4 weeks. After that, the rats were fasted overnight. STZ (Sigma, St. Louis, MO, USA) was freshly prepared in a 0.05 M citrate buffer (pH 4.5), and a single intraperitoneal (i.p.) injection of 35 mg kg^−1^ was used to induce T2DM in each rat. The blood glucose level was monitored every 3 days using an Accu-Chek blood glucose meter (Roche Diagnostics, Basel, Switzerland). Stable hyperglycemia was established in the rats (denoted by blood glucose levels ≥ 200 mg/dl (15 mM)) seven days after the STZ injection.

Fourteen rats acted as the control where they were injected with citrate buffer. After that, STZ at 35 mg kg^−1^ was injected intraperitoneally [18]. Rats with plasma glucose levels of 150–280 mg/dl were selected for the following experiment.

### 2.5. Experimental Design

The experimental model involved six groups with 7 rats in each as follows: control group: nondiabetic rats received saline; GCWE group: nondiabetic rats received green coffee water extract (100 mg kg^−1^ day^−1^); diabetic group: diabetic rats received saline; diabetic-metformin group: the rats in this group were orally given a metformin dose of 200 mg kg^−1^; diabetic-GCWE-50 group: diabetic rats orally treated with green coffee (50 mg kg^−1^ day^−1^; equivalent to one cup consumed by a man per day); and diabetic-GCWE-100 group: diabetic rats treated orally with green coffee (100 mg kg^−1^ day^−1^; equivalent to two cups consumed by a man per day). All the rats received their treatment daily for 28 days.

### 2.6. Sampling

At the end of the experiment, the rats were sacrificed by cervical decapitation with 300 mg kg^−1^ pentobarbital (i.p.) after 24 h treatment. For obtaining serum, the collected blood samples were incubated at room temperature for 30 min followed by centrifugation at 3000 × *g* for 10 min. The obtained serum was stored at −80°C for further biochemical assays. The testes were collected and weighed, a part was fixed in 10% buffered formalin for histopathological investigations, and another part was kept at -80°C for performing biochemical studies and molecular techniques.

### 2.7. Hormone Analysis

Specific assay kits were intended for measuring the levels of plasma testosterone, luteinizing hormone (LH), and follicle-stimulating hormone (FSH) by ELISA according to the manufacturer's protocols. The optical density (OD) was measured at 450 nm with a reference wavelength of 620–630 nm using a spectrophotometer (BioTek Inc., Germany).

### 2.8. Investigation of Oxidative Stress Markers

Grinding and homogenizing of testes was performed by mixing with 10 mM phosphate buffer (pH 7.4). The supernatant was prepared from the testes by centrifugation of homogenate for 10 min (3000 × *g*) at 4°C. The lipid peroxide (LPO) level was measured as described by Ohkawa et al. [19]. The protein carbonyl (PCO) content was determined by the 2,4-dinitrophenylhydrazine test with slight modifications [20]. The nitric oxide (NO) level was detected according to the method of Green et al. [21] that used the Griess reagent. The activity of reduced glutathione (GSH) was investigated using the method of Ellman [22]. The superoxide dismutase (SOD) level was evaluated following the standard technique described by Nishikimi et al. [23]. The catalase (CAT) activity was demonstrated following the method described by Aebi [24]. The activities of glutathione peroxidase (GPx) and glutathione reductase (GR) were determined according to the procedure described by Paglia and Valentine [25] and De Vega et al. [26], respectively.

### 2.9. Measurement of Inflammatory Markers

The levels of tumor necrosis factor- (TNF-) *α* and interleukin- (IL-) 1*β* were detected in testes by kits of ELISA obtained from R&D Systems and Thermo Fisher Scientific, respectively, following the manufacturers' instructions.

### 2.10. Determination of Apoptotic Protein Levels

The levels of Bcl-2, Bax, and caspase-3 proteins in testicular tissues were determined by commercial ELISA kits obtained from Cusabio Life Sciences (Wuhan, China) following the instructions of the manufacturer.

### 2.11. Quantitative Real-Time PCR Analysis

RNA was isolated from freshly removed testicular tissues using the TRIzol reagent (Qiagen, Germantown, MD, USA) following the instructions of the manufacturer. The RNA concentration was determined using NanoDrop, and then, it was reverse-transcribed into cDNA by using a kit of RevertAid™ H Minus Reverse Transcriptase supplied by Fermentas, Thermo Fisher Scientific Inc., Canada, according to the manufacturer's protocol. An SYBR Green PCR kit (Qiagen, Germany) was used to determine mRNA levels of *Sod2*, *Cat*, *Gpx1*, *Gsr*, *Bcl2*, *Bax*, and *Casp3*. Quantitative PCR was performed in triplicate on a ViiA™ 7 PCR system (Applied Biosystems, USA). The relative levels of mRNA were calculated by the 2^-*ΔΔ*Ct^ method, which was normalized to the mRNA level of the *Actb* housekeeping gene. Primer sequences are shown in Table 1.

### 2.12. Histological Procedures

The right testis from rats was cut into smaller parts and fixed in 10% neutral buffered formalin for 24 h at room temperature. 5 *μ*m thick sections were cut from the collected paraffin blocks, then deparaffinized in xylene. Sections were counterstained with hematoxylin and eosin and examined by a Nikon Eclipse E200-LED microscope (Tokyo, Japan) with various magnifications to record any histopathological alterations. Sections were evaluated according to the modified Johnsen scoring system [27] as follows: 1: absence of seminiferous epithelium and tubular sclerosis; 2: absence of germinal cells, while Sertoli cells were presented; 3: only spermatogonia; 4: no spermatids, few spermatocytes, and arrest of spermatogenesis at the primary spermatocyte stage; 5: absence of spermatozoa and spermatids and frequent spermatocytes; 6: absence of spermatozoa and late spermatids, arrest of spermatogenesis at the spermatid stage, and disturbance of spermatid differentiation; 7: absence of spermatozoa and late spermatids and many early spermatids; 8: less than 5 spermatozoa/tubules and few late spermatids; 9: spermatogenesis slightly impaired, frequent late spermatids, and disorganized epithelium; and 10: full spermatogenesis observed.

### 2.13. Statistical Analysis

Statistics were done by utilizing SPSS software, and results were represented as mean ± SD. The difference between multiple groups was evaluated by one-way ANOVA, with *p* < 0.05 considered significant.

## 3. Results

### 3.1. Total Phenolic and Flavonoid Content of GCWE

The obtained data revealed that the TP level was 131.3 mg GAE/g GCWE DW and the TF level was 40.7 mg QE/g GCWE DW. These obtained results were consistent with the previous studies of Jeszka-Skowron et al. [28] and Madhava Naidu et al. [29].

### 3.2. Effect of Green Coffee on Testes' Weights

**Figure 1:**
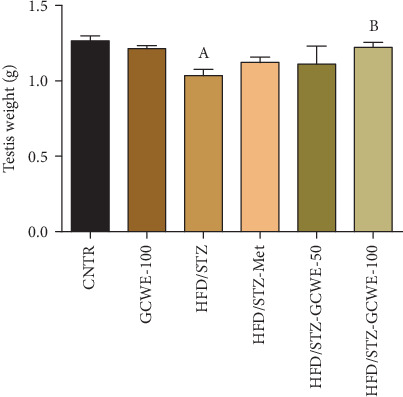
Testis weight following green coffee water extract (50 and 100 mg kg^−1^) or metformin (200 mg kg^−1^) in high-fat diet/streptozotocin-induced diabetes in rats. Data are expressed as mean ± SD (*n* = 7). A. The statistical significance relative to that of the control (CNTR) group at *p* < 0.05. B. The statistical significance relative to that of the diabetic (HDF/STZ) group at *p* < 0.05.

As shown in Figure 1, a significant reduction in the testes' weights was observed in the HFD/STZ group (*p* < 0.05) compared to the control rats. However, weights of testes were found to be significantly increased in groups treated with green coffee or metformin compared to the diabetic rats (*p* < 0.05). Interestingly, the two doses (50 mg kg^−1^ and 100 mg kg^−1^) of green coffee groups showed a significant change in the testes' weights suggesting that the green coffee administration could constrain the loss in rat testis weight caused by HFD and STZ depending on a dose-dependent manner.

### 3.3. Effect of Green Coffee on Sex Hormone Levels

**Figure 2:**
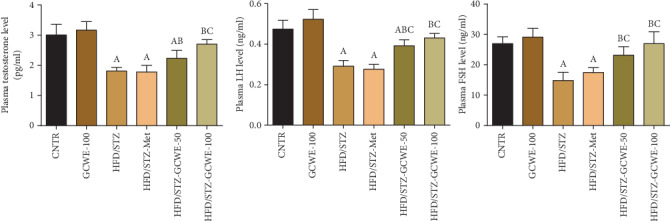
Plasma testosterone, luteinizing hormone (LH), and follicle-stimulating hormone (FSH) following green coffee water extract (50 and 100 mg kg^−1^) or metformin (200 mg kg^−1^) in high-fat diet/streptozotocin-induced diabetes in rats. Data are expressed as mean ± SD (*n* = 7). A. The statistical significance relative to that of the control group (CNTR) at *p* < 0.05. B. The statistical significance relative to that of the diabetic group (HDF/STZ) at *p* < 0.05. C. The statistical significance relative to that of the diabetes-metformin-treated (HDF/STZ-Met) group at *p* < 0.05.

As shown in Figure 2, comparing the levels of sex hormones between the control group and the diabetic group, STZ administration significantly reduced the levels of LH, FSH, and testosterone (*p* < 0.05). Interestingly, compared to the STZ group, a significant increase (*p* < 0.05) in the levels of these hormones was observed in the group treated with green coffee. Meanwhile, no changes in the levels of LH and FSH along with a significant elevation of testosterone levels were detected in metformin-treated animals compared to those of the STZ diabetic group. Notably, a significant reduction in levels of testosterone and LH was observed in green coffee- (50 mg kg^−1^) and metformin-treated rats compared to the control levels.

### 3.4. Effect of Green Coffee on STZ-Induced Testicular Oxidative Stress

**Figure 3:**
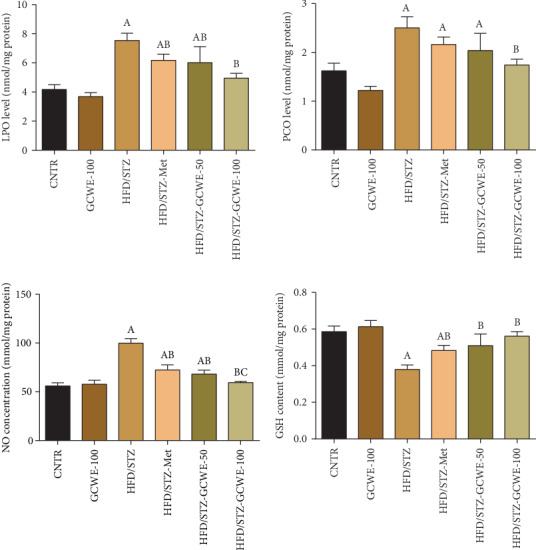
Testicular lipid peroxide (LPO), protein carbonyl (PCO), nitric oxide (NO), and glutathione (GSH) following green coffee water extract (50 and 100 mg kg^−1^) or metformin (200 mg kg^−1^) in high-fat diet/streptozotocin-induced diabetes in rats. Data are expressed as mean ± SD (*n* = 7). A. The statistical significance relative to that of the control group (CNTR) at *p* < 0.05. B. The statistical significance relative to that of the diabetic group at *p* < 0.05. C. The statistical significance relative to that of the diabetes-metformin-treated (HDF/STZ-Met) group at *p* < 0.05.

**Figure 4:**
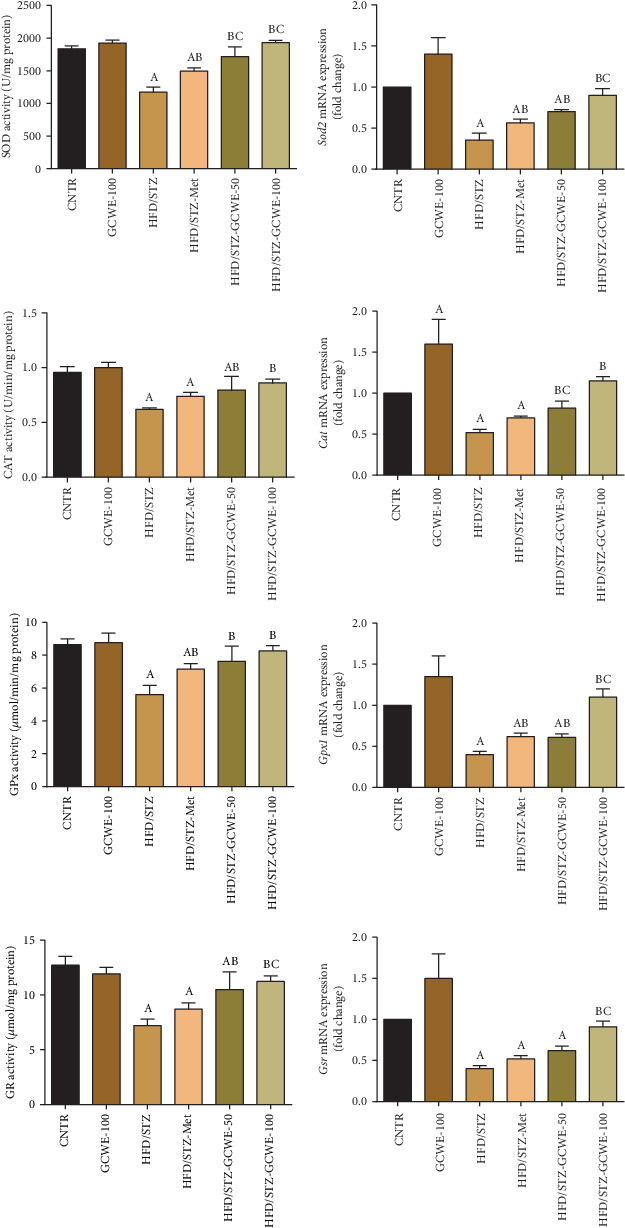
Testicular superoxide dismutase (SOD), catalase (CAT), glutathione peroxidase (GPx), and glutathione reductase (GR) following green coffee water extract (50 and 100 mg kg^−1^) or metformin (200 mg kg^−1^) in high-fat diet/streptozotocin-induced diabetes in rats. Biochemical data are expressed as mean ± SD (*n* = 7). mRNA expression results are recorded as mean ± SD of three assays in duplicate referenced to *Actb* and represented as fold changes (log2 scale) as compared with the mRNA levels of the control group. A. The statistical significance relative to that of the control group (CNTR) at *p* < 0.05. B. The statistical significance relative to that of the diabetic group (HDF/STZ) at *p* < 0.05. C. The statistical significance relative to that of the diabetes-metformin-treated (HDF/STZ-Met) group at *p* < 0.05.

Administration of STZ resulted in oxidative stress of testicular tissues as indicated by increasing levels of prooxidants such as LPO, PCO, and NO and decreasing GSH content (Figure 3). Reduced activities of antioxidants such as SOD, CAT, GPx, and GR were also observed (Figure 4). The mRNA expression of *Sod2*, *Cat*, *Gpx1*, and *Gsr* was significantly downregulated (*p* < 0.05) in the STZ diabetic group compared to the control group. Conversely, green coffee (50 mg kg^−1^ and 100 mg kg^-l^), as well as metformin treatments, exerted antioxidant effects by increasing the protein and mRNAs expressions of *Sod2*, *Cat*, *Gpx1*, and *Gsr* together with a significant decline in the levels of LPO, PCO, and NO compared to the HFD/STZ group. Notably, a significant reduction at the protein and mRNA levels of *Sod2*, *Cat*, and *Gpx1* was recorded; however, levels of LPO, PCO, and NO were significantly upregulated in testicular tissues of both the green coffee- (50 mg kg^−1^) and metformin-treated rats compared to the control rats.

### 3.5. Effect of Green Coffee Administration on STZ-Mediated Testicular Inflammation

**Figure 5:**
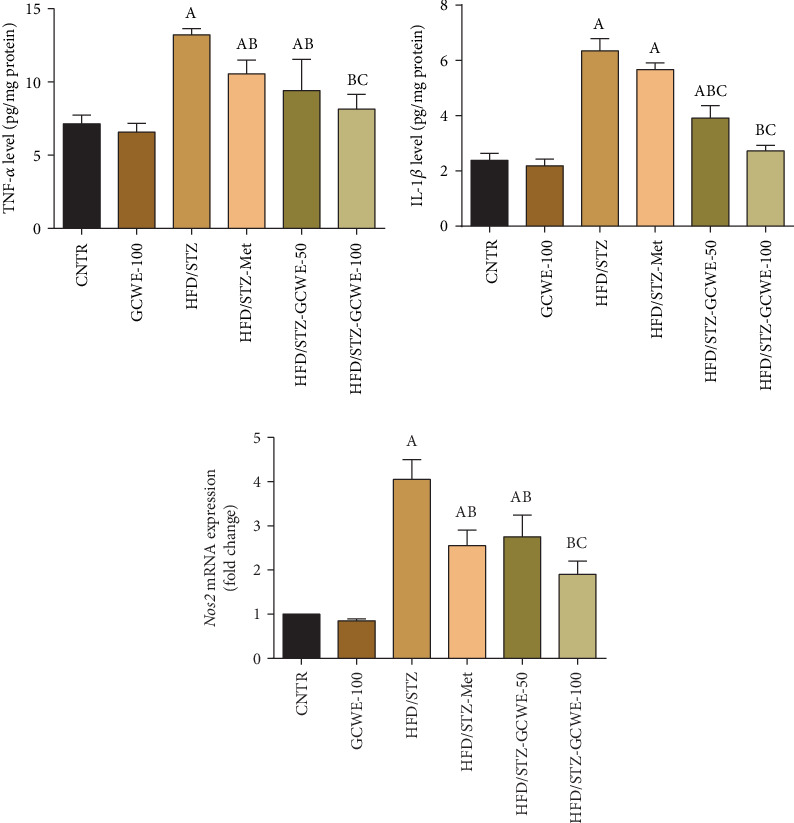
Testicular tumor necrosis factor- (TNF-) *α*, interleukin- (IL-) 1*β*, and inducible nitric oxide synthase (*Nos2*) following green coffee water extract (50 and 100 mg kg^−1^) or metformin (200 mg kg^−1^) in high-fat diet/streptozotocin-induced diabetes in rats. ELISA data are expressed as mean ± SD (*n* = 7). mRNA expression results are recorded as mean ± SD of three assays in duplicate referenced to *Actb* and represented as fold changes (log2 scale) as compared with the mRNA levels of the control group. A. The statistical significance relative to that of the control group (CNTR) at *p* < 0.05. B. The statistical significance relative to that of the diabetic group (HDF/STZ) at *p* < 0.05. C. The statistical significance relative to that of the diabetes-metformin-treated (HDF/STZ-Met) group at *p* < 0.05.

STZ administration mediated an inflammatory reaction through an increase (*p* < 0.05) of IL-1*β* and TNF-*α* at the protein level and an increase of *Nos2* at the mRNA level in testes of diabetic animals compared to the control group (Figure 5). Conversely, green coffee and metformin treatments reduced testicular inflammation as demonstrated by a significant decline in the protein levels of IL-1*β* and TNF-*α* compared to the HFD- and STZ-treated rats (*p* < 0.05).

### 3.6. Effect of Green Coffee on the Apoptosis-Related Markers in the Testes

**Figure 6:**
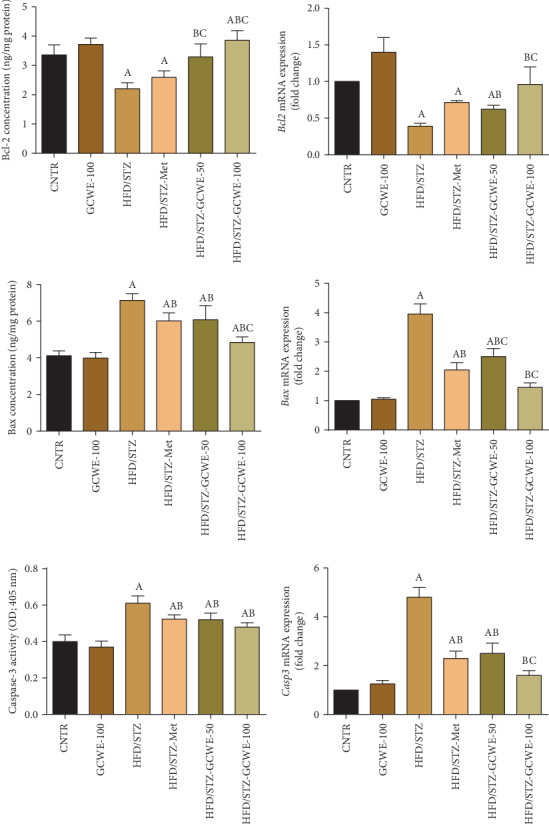
Testicular apoptotic-related protein levels and expressions following green coffee water extract (50 and 100 mg kg^−1^) or metformin (200 mg kg^−1^) in high-fat diet/streptozotocin-induced diabetes in rats. ELISA data are expressed as mean ± SD (*n* = 7). mRNA expression results are recorded as mean ± SD of three assays in duplicate referenced to *Actb* and represented as fold changes (log2 scale) as compared with the mRNA levels of the control group. A. The statistical significance relative to that of the control group (CNTR) at *p* < 0.05. B. The statistical significance relative to that of the diabetic group (HDF/STZ) at *p* < 0.05. C. The statistical significance relative to that of the diabetes-metformin-treated (HDF/STZ-Met) group at *p* < 0.05.

In the current study, in parallel to the results of the control group, STZ administration significantly promoted apoptosis in the testicular tissue of rats as shown by the elevation of the proapoptotic protein levels of Bax and caspase-3 and reduction of the Bcl-2 level (*p* < 0.05) (Figure 6). qRT-PCR results were in line with the protein results. The *Bax* and *Casp3* mRNA expression results were upregulated, while *Bcl2* expression was downregulated in the diabetic treated rats compared to the control group (Figure 6). In contrast, green coffee and metformin treatments enhanced the antiapoptotic pathway through the upregulation of *Bcl2* and downregulation of *Bax* and *Casp3* compared to the diabetic group. However, *Bax* and *Casp3* showed a significant rise in their levels in both green coffee- and metformin-treated rats compared to the control group.

### 3.7. Histopathological Alterations in the Testes

**Figure 7:**
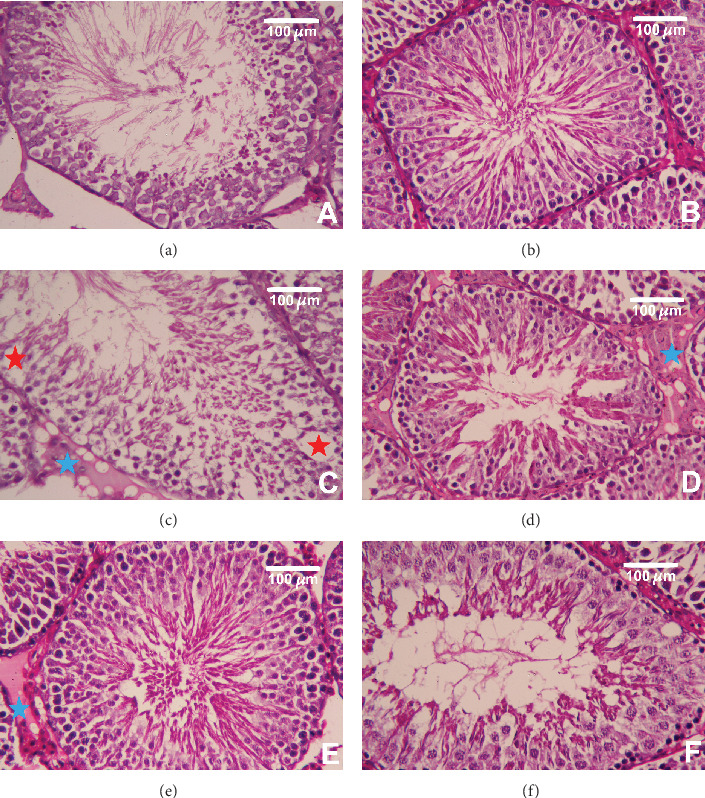
Histopathological alterations of testicular tissue following green coffee water extract (50 and 100 mg kg^−1^) or metformin (200 mg kg^−1^) in high-fat diet/streptozotocin-induced diabetes in rats. Normal testicular architecture in the control and green coffee water extract including normal seminiferous tubule structure with normal spermatogenesis (a, b). In contrast, high-fat diet/streptozotocin diabetic rats exhibited fat droplets between the tubules (blue star) and degeneration of spermatogenic cells alongside vacuolated patches within the seminiferous tubules (red star) (c). However, pretreatment with metformin or green coffee water extract (50 and 100 mg kg^−1^) reversed the histological alterations in response to high-fat diet/streptozotocin diabetes (d–f). Scale bar = 100 *μ*m.

**Figure 8:**
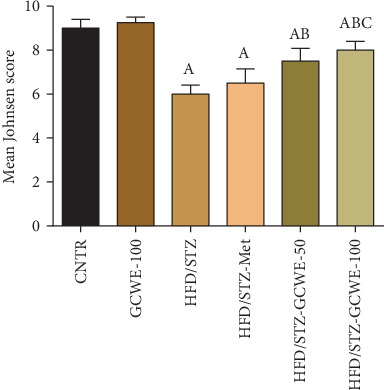
Effect of green coffee water extract (50 and 100 mg kg^−1^) or metformin (200 mg kg^−1^) on the Johnsen score in high-fat diet/streptozotocin-induced diabetes in rats. Data are expressed as mean ± SD (*n* = 7). A. The statistical significance relative to that of the control group (CNTR) at *p* < 0.05. B. The statistical significance relative to that of the diabetic group at *p* < 0.05. C. The statistical significance relative to that of the diabetes-metformin-treated (HDF/STZ-Met) group at *p* < 0.05.

In Figure 7, both the testicular tissues of the control and green coffee-treated groups showed typical testicular architecture including normal seminiferous tubule structure with normal spermatogenesis (Figures 7(a) and 7(b), respectively). In the HFD/STZ group, a decrease in the number of spermatozoa, poor organization, and obvious degeneration of spermatogenic cells were observed alongside vacuolated patches and fat droplets within the seminiferous tubules (Figure 7(c)). Animals treated with metformin or green coffee (low and high doses) showed improvement in the pathological changes in rats' testes induced by HFD and STZ as shown by the normal architecture of seminiferous tubules (Figures 7(d)–7(f), respectively). Interestingly, there was no significant difference between the CGWE-100 and control groups in all our experiments showing that green coffee extract treatment alone has no effect on the normal testicular tissues. Furthermore, the data obtained from the Johnsen scoring system were consistent with these morphological observations. The HFD/STZ group had low scores on the Johnsen scoring system, which indicated the presence of degenerative seminiferous tubules. However, CGWE-treated rats, even though the levels did not reach completely back to control values, showed significant improvement compared to the HFD/STZ group in both the low and high doses (Figure 8).

## 4. Discussion

In the current study, HFD and STZ were used for the induction of diabetes mellitus in male rats. It was previously shown that HFD causes insulin insensitivity and then low-dose STZ causes islet cell destruction [30]. Enhanced oxidative stress [2], decreased antioxidant capacity, inflammation [31], and apoptosis [32] have been considered to be the most important mechanisms not only in HFD- but also in STZ-induced testicular dysfunction [3].

In the present study, the significant weight loss of the testis and the reduced levels of T, FSH, and LH detected in diabetic rats were consistent with many previous works [33, 34]. These adverse effects may have resulted from excessive oxidative stress and apoptosis in the tissues of the testes that dramatically give rise to testicular dysfunction [35, 36].

Additionally, it was indicated that spermatogenesis and sperm functions are strongly related to the hypothalamic-pituitary-gonadal axis. Alterations in GnRH signaling affect both the LH and FSH secretion that stimulates steroidogenesis and spermatogenesis, respectively, in the Leydig and Sertoli cells [37]. Sertoli cells have a primary role in spermatogenesis wherein utilizing energy by other pathways than glucose metabolism may alter their organelle structures and result in cellular damage and incomplete maturation process [38]. Importantly, green coffee was capable of attenuating testicular damage by diminishing the loss in weight of the testis and raising the concentrations of sex hormones suggesting a protective ability of green coffee. Previously, Wedick et al. [39] found that coffee consumption significantly elevated total testosterone and declined both the total and free estradiol after 28 days which suggests that caffeine may act as an aromatase inhibitor.

SOD, CAT, GSH, and GPx are antioxidant enzymes that represent the main defense mechanism against reactive oxygen species (ROS) generation in the organism where they are responsible for scavenging the toxic intermediate of incomplete oxidation. In normal conditions, these enzymes are deployed to abolish the excessive production of ROS and reduce the damage caused by oxidants. Nevertheless, in hyperglycemia, the antioxidant enzymes undergo glycosylation leading to their decreased activities and eliciting oxidative damage [40].

In our study, a decline in the activities of SOD, CAT, GR, and GPx was detected in the HFD and STZ diabetic rats which could be due to the overproduction of ROS that overwhelms antioxidant mechanisms. This disturbance of antioxidant capability might be responsible for the stimulation of testicular oxidative injury as was previously indicated by Wang et al. [33]. Oxidative stress induced by hyperglycemia in the present experimental model was similar to that indicated by Ghosh and Mukherjee [35]. Furthermore, Zhao et al. [41] indicated that hyperglycemia causes excessive accumulation of ROS that accelerates cellular oxidative injury and endoplasmic reticulum stress.

It was indicated that ROS causes deleterious alterations in the male reproductive system [42]. Such alterations include testicular complications like abnormal spermatogenesis and oligospermia along with a decreased level of reproductive hormones [43]. Moreover, spermatozoa were found to be more susceptible to ROS production which could reduce sperm motility causing infertility [3].

Lipid peroxides can be formed via the enzymatic and/or nonenzymatic mechanisms. Indeed. It was previously shown that high glycemic levels and ROS generation are the leading causes for the initiation of lipid peroxidation which followed the damage of cellular compartments and function [33]. It has been also shown that malondialdehyde (MDA) is a final product of lipid peroxidation in the cells. The increased MDA level detected in the diabetic group of the present results was consistent with those previously reported by Wang et al. [33] and Zhao et al. [44]. The same studies demonstrated that a rise in free radical production causes overproduction of MDA that resulted in the imbalance of antioxidant integrity.

In our study, the increased production of LPO and decreased content of GSH, SOD, GR, and GPx were obviously restored by the administration of green coffee, suggesting a clear antioxidant activity of the green coffee extract that was in line with those demonstrated in several studies [9, 10]. Coffee contains high amounts of the chlorogenic, caffeic, and ferulic acids. Pharmacological activities of those ingredients are related to its high antioxidant activity, in particular, its capacity to suppress lipid peroxidation in a biological system [45].

Inflammation is widely accepted to be responsible for the pathogenesis of hyperglycemia and its complications [7]. It was, also, stated that the levels of both TNF-*α* and IL-1*β* have a direct impact on the initiation and complications of diabetes [46]. Similarly, the TNF-*α* and IL-1*β* levels were significantly elevated in the testes of diabetic rats. The inflammatory reaction shown in the present study can be explained by the following: ROS resulted in the oxidation of protein and lipids in some cellular structures, which causes cell injury [47] by triggering ER (endoplasmic reticulum) stress, debilitating normal mitochondrial function, and disrupting the DNA. These complications enhance NF-*κ*B-mediated inflammation in conjunction with the increased levels of proinflammatory mediators such as TNF-*α* and IL-1*β* [32]. The levels of inflammatory cytokines decreased significantly after treatment with green coffee, suggesting that green coffee can exert an anti-inflammatory effect by decreasing the IL-1*β* and TNF-*α* levels which has been also suggested by numerous studies [10, 48]. Our results were in agreement with Pergolizzi et al. [49] who found that the anti-inflammatory effect of green coffee may contribute to the antiradical effect. Furthermore, Budryn et al. [50] found that the active ingredients of coffee prevent inflammation through the suppression of oxidative pressure and the inactivation of the TLR4/MyD88/NF-*κ*B signaling pathway in the liver of rats intoxicated with carbon tetrachloride.

Apoptosis is a genetically organized pathway for cell death under different pathophysiological conditions. Apoptotic pathways are associated with caspase-mediated degradation of DNA by DNases, giving it a ladder-like pattern [51]. The intrinsically dependent or mitochondrial pathway is one of the apoptotic pathways that involves crosstalk between proapoptotic members of the Bcl-2 family, cytochrome c, and caspases [52].

In our study, STZ diabetic rats exhibited an increased expression of the proapoptotic Bax and caspase-3 along with the decreased level of antiapoptotic Bcl-2. Similarly, a previous study indicated that hyperglycemia induced apoptosis through the disruption in the balance between Bax and Bcl-2 proteins. This imbalance may induce the discharge of cytochrome c from the mitochondrial matrix to the cytosol resulting in the increased concentration of cleaved caspase-3 that promotes degradation of DNA by DNases [32].

Sperm production from the germinal cell in the testes takes place by keeping a balance between cell proliferation and cell death [53]. Apoptosis observed in the current work may be responsible for the testicular dysfunction as was mentioned by Ghosh and Mukherjee [35] and Wang et al. [34]. Similarly, many studies have indicated that not only HFD but also the injection of STZ led to excessive apoptosis of testicular germ cells that resulted in abnormal spermatogenesis and low concentration of spermatozoa with poor morphology [34, 54].

Oxidative stress may be considered a potent inducer of cell apoptosis observed in this study as was previously mentioned by Yang et al. [55]. However, green coffee treatment significantly inhibited STZ-induced apoptosis of testicular cells by decreasing the expression of the Bax and caspase-3 together with the increasing level of Bcl-2. These observations suggested that suppressing the mitochondrial-dependent pathway of apoptosis might be an important pathway through which green coffee prevents abnormal spermatogenesis induced by HFD and STZ.

We conclude that green coffee treatment attenuated testicular oxidative damage which modulates inflammatory reaction and apoptotic-related pathways. Green coffee exerts its effects by increasing the antioxidant activity and suppressing inflammatory response and the apoptotic pathway in the testes. Interestingly, metformin also has similar effects on green coffee on all the parameters investigated in this study. This suggests that green coffee could be used to substantiate metformin in the treatment of diabetes-induced testicular dysfunction.

## Figures and Tables

**Table 1:** Primer sequences of genes analyzed in real-time PCR.

Name	Accession number	Sense (5′—3′)	Antisense (5′—3′)
*Actb*	NM_031144.3	GCAGGAGTACGATGAGTCCG	ACGCAGCTCAGTAACAGTCC
*Sod2*	NM_001270850.1	AGCTGCACCACAGCAAGCAC	TCCACCACCCTTAGGGCTCA
*Cat*	NM_012520.2	TCCGGGATCTTTTTAACGCCATTG	TCGAGCACGGTAGGGACAGTTCAC
*Gpx1*	NM_017006.2	CGGTTTCCCGTGCAATCAGT	ACACCGGGGACCAAATGATG
*Gsr*	NM_053906.2	TGCACTTCCCGGTAGGAAAC	GATCGCAACTGGGGTGAGAA
*Bcl2*	NM_016993.1	CTGGTGGACAACATCGCTCTG	GGTCTGCTGACCTCACTTGTG
*Bax*	NM_017059.2	GGCGAATTGGCGATGAACTG	ATGGTTCTGATCAGCTCGGG
*Tnfα*	XM_008772775.2	AGAACTCAGCGAGGACACCAA	GCTTGGTGGTTTGCTACGAC
*Il1β*	NM_031512.2	GACTTCACCATGGAACCCGT	GGAGACTGCCCATTCTCGAC
*Nos2*	NM_012611.3	GTTCCTCAGGCTTGGGTCTT	TGGGGGAACACAGTAATGGC

The abbreviations of the genes: *Actb*: *β*-actin; *Sod2*: manganese-dependent superoxide dismutase (MnSOD); *Cat*: catalase; *Gpx1*: glutathione peroxidase; *Gsr*: glutathione reductase; *Bcl2*: B-cell lymphoma 2; *Bax*: Bcl-2-like protein 4; *Tnfα*: tumor necrosis factor alpha; *Il1β*: interleukin-1 beta; *Nos2*: nitric oxide synthase 2.

## Data Availability

All relevant data are within the paper.
